# Estimating the Relative Sociolinguistic Salience of Segmental Variables in a Dialect Boundary Zone

**DOI:** 10.3389/fpsyg.2016.01163

**Published:** 2016-08-15

**Authors:** Carmen Llamas, Dominic Watt, Andrew E. MacFarlane

**Affiliations:** Department of Language and Linguistic Science, University of YorkYork, UK

**Keywords:** salience, perception, borders, isogloss, indexicality, nationality, accent, dialect

## Abstract

One way of evaluating the salience of a linguistic feature is by assessing the extent to which listeners associate the feature with a social category such as a particular socioeconomic class, gender, or nationality. Such ‘top–down’ associations will inevitably differ somewhat from listener to listener, as a linguistic feature – the pronunciation of a vowel or consonant, for instance – can evoke multiple social category associations, depending upon the dialect in which the feature is embedded and the context in which it is heard. In a given speech community it is reasonable to expect, as a consequence of the salience of the linguistic form in question, a certain level of intersubjective agreement on social category associations. Two metrics we can use to quantify the salience of a linguistic feature are (a) the speed with which the association is made, and (b) the degree to which members of a speech community appear to share the association. Through the use of a new technique, designed as an adaptation of the Implicit Association Test, this paper examines levels of agreement among 40 informants from the Scottish/English border region with respect to the associations they make between four key phonetic variables and the social categories of ‘Scotland’ and ‘England.’ Our findings reveal that the participants exhibit differential agreement patterns across the set of phonetic variables, and that listeners’ responses vary in line with whether participants are members of the Scottish or the English listener groups. These results demonstrate the importance of community-level agreement with respect to the associations that listeners make between social categories and linguistic forms, and as a means of ranking the forms’ relative salience.

## Introduction

The study of the salience of speech sounds and other linguistic units can be approached in a diversity of ways, each based on different sets of assumptions about the nature and relative magnitude of the effect that an external stimulus has on the subject who is exposed to it. For some purposes it may be appropriate to focus on what salience means in terms of differences in the response sensitivity of the human peripheral auditory system, or to investigate how patterns of neuronal activation reveal inequalities in the prominence of certain speech stimuli relative to others or to background noise. We will henceforth use the term ‘salience’ to refer to that property of a spoken form which causes listeners to respond to the form in such a way as to indicate that it encodes information about the (presumed) social characteristics and/or geographical origins of the speaker, alongside the linguistic functions that the form simultaneously fulfills (e.g., to help to distinguish the word in which it appears from other plausible candidate words): sociolinguistic salience, in other words. Forms with high salience are, according to this definition, argued to index social information more unequivocally than do forms with lower salience. Variation in the directness of the mapping of sounds to speakers’ non-linguistic characteristics means that when we test the association between the form and the social category it evokes, listeners are likely to respond faster and more consistently to high salience forms than they are to low salience forms.

To deduce the relative strength of a phonetic form’s sociolinguistic salience we must establish that the form does indeed function as a vehicle for social meaning. Also, for the association to be meaningful in terms of its capacity to index social information, the association should be the property of the group rather than just the individual, as such meaning is shared meaning. The association, therefore, must be one that listeners will generally agree upon.

‘Top–down’ associations of this type will inevitably differ between listeners, so strictly one-to-one relationships between phonetic forms and social group associations are unlikely to exist. Linguistic features carry multiple social category associations depending on the variety in which the features are embedded, the listeners’ experience of the variety, and the context in which the features are heard (see further Niedzielski, 1999; Clopper and Pisoni, 2004; Johnstone and Kiesling, 2008; McGowan, 2015). As a result, any phonetic form may potentially index multiple social factors, as different listeners may associate it with different social groups.

As a testbed for the above claims we focus upon the border zone between Scotland and England that our previous research in the area (Llamas, 2010; Watt et al., 2014b) has shown us to be a region in which the prevalence of linguistic markers of ingroup and outgroup status is of particular significance. Consensus levels among community members with respect to social category associations with phonetic forms are quantified in the current study via an innovative adaptation of the Implicit Association Test (IAT; Greenwald et al., 1998). The new Social Category Association Test (SCAT) we present here allows the strength of association to be measured through analysis of response times, with faster responses implying a higher degree of certainty on the listener’s part about the association, and slower ones demonstrating a level of hesitancy from which we can infer that the association is weaker and less direct.

As well as looking at shared agreement on social meaning across the border zone as a whole, we investigate differences in the responses gathered from inhabitants of communities on either side of the border and at its ends, where the border meets the coast. Age- and gender-related differences are also examined. We begin by considering the role of salience and social meaning in how languages vary synchronically and change over time, before outlining the benefits of utilizing border zones – and the particular border zone under investigation in our study – as test sites for the operationalization of salience as an observable quantity. After examination of the results, we assess the extent to which we can propose differing degrees of salience among segmental forms, based on community-level agreement on the forms’ social category associations.

### Salience and Social Meaning

The salience of a variable linguistic feature, from a sociolinguistic point of view, relates to the level of awareness that speakers have of that variable, which in turn is connected to the social meanings that become attached to its variants. According to Rácz (2013), salience is an essential predictor of whether an *indicator* (a linguistic unit which varies non-randomly according to speakers’ social characteristics) will become a *marker* (a feature of which speakers are aware to the extent that they adjust their use of it in line with the amount of attention they are paying to their speech; see further Labov, 1972, pp. 178–180). Increasing awareness of a marker may lead to it becoming a *stereotype*, in Labov’s taxonomy, or acquiring ‘third order indexicality,’ as per Silverstein’s (2003) model. However, by the time features become the topic of overt social comment, they may have become recessive in actual speech production. An example of a form which has attained the status of a stereotype is the apical trilled /r/ in Scottish English, which is popularly associated very closely with that variety, but which in fact occurs in modern Scottish English only infrequently.

The explanatory potential of salience as a motivating factor in language change has long been acknowledged, though the factors involved in a variable becoming salient are still much debated. Trudgill (1986, p. 11) describes a set of testable criteria, comprised of both external (language-extrinsic, social) and internal (language-intrinsic) factors, according to which salience can be attributed to forms in interactional situations. External factors include whether or not a variable is currently undergoing change, and the degree of overt stigmatization of its variants. Stigmatization of this kind often relates to whether a high-prestige form is represented in orthography; an example from British English is the long-standing stigma attached to /h/-dropping in content words such as *hat* or *house*. Internal factors include the maintenance of phonological contrast and the phonetic distance between the variants of a variable, whereby variants that are highly distinct from one another are more salient. Certain features, Trudgill claims, possess ‘extra-strong salience’ thanks to their ‘overly strong’ association with particular accents or dialects. Forms of this sort are so closely associated with certain varieties, perhaps to the point of iconicity, that they tend to inhibit accommodation in dialect contact situations (see Llamas et al., 2009; Watt et al., 2010 for discussion of accommodation effects and salience in the border context under investigation in the present study).

Kerswill and Williams (2002, p. 83) criticize Trudgill’s criteria, arguing for the inclusion of extra-linguistic cognitive, social-psychological and pragmatic factors in the pool of factors contributing to salience. They do so as a way of attempting to eliminate the circularity inherent in definitions of salience which claim that forms are salient by virtue of language users being more highly aware of them than they are of other forms. The extra-linguistic factors listed above are, according to Kerswill and Williams (2002, p. 105), ‘ultimately the cause of salience.’ Any operationalization of the notion of salience must, Kerswill and Williams assert, involve consideration of three components: (1) some patterning of language change or language variation for which the explanation may lie in the salience of the feature in question; (2) language-internal factors, such as the maintenance of phonological contrast; and (3) extra-linguistic cognitive, pragmatic, interactional, social-psychological, and socio-demographic factors (Kerswill and Williams, 2002, p. 105). Awareness on the researcher’s part of the subjective evaluation of forms and their embedding in linguistic structure is also a crucial element, given that these phenomena are subject to change within and beyond the speech community.

The primacy of social factors argued for by Kerswill and Williams (2002) is challenged by Hollmann and Siewierska (2006), who contend that cognitive perceptual factors are paramount. Through an examination of the Lancashire dialect, they propose that linguistic forms are free from social values when the forms first come into existence, and it is only after the forms have emerged that social forces start working on them (Hollmann and Siewierska, 2006, p. 27). They identify properties such as token frequency and transparency of the form-meaning relation as examples of the perceptual-cognitive factors they have in mind. Hollmann and Siewierska (2006, p. 27) concede that social factors play an important role in the process by which social values come to be attached to forms, but conclude that ‘ultimately it is the cognitive-perceptual constraints that make a form more or less liable to becoming subject to social evaluation and patterning.’

A similar position is adopted by Rácz (2013), who distinguishes between *cognitive salience* and *sociolinguistic salience*. The former, he argues, stems from the perceived difference between the transitional probability patterns of the realization of the variable in one dialect as opposed to another, which leads to listeners’ ‘surprisal’ and noticing of the variable. A form accrues sociolinguistic salience, by contrast, if it is mobilized for the purposes of social indexation (Rácz, 2013, p. 10). One of the case studies drawn upon by Rácz in his examination of salience in sociolinguistics is rhoticity (r-fulness) in Scotland. This has obvious relevance to the present study, as we shall see. Rácz (2013, p. 21) argues that rhoticity is a phonetically fine-grained process, and that the extent of phonetic variation in coda /r/ is such that it prevents listeners from targeting the feature as a reliable carrier of social indexation. Rácz also claims that the salience of features in a given speech community can be determined by a number of independent measures, proposing that the ‘best tools are attitude studies, which clearly show whether listeners associate the presence versus absence of a variant with a particular geographical location or social stratum’ (Rácz, 2013, p. 8). We will suggest in the current study that, on the basis of overt comments drawn from attitudinal data collected from our informants, the presence or absence of coda /r/ in words such as *car* or *farm* is seen as a key indicator of whether a talker is from Scotland or from England. Surprisingly, however, we will see that in the perceptual testing strand of the project the expected association of rhoticity with Scotland turns out not to be a robust one.

Though we do not deny its central relevance to the study of perceptual salience, we are less concerned here with the *process* by which salience becomes attached to a linguistic form than we are with how to establish the degree of salience that that feature possesses. We would argue that a form’s salience is linked both to its capacity to index social meaning and the functions that it actually fulfills in this respect. It is probably true to say that, in terms of their potential for perceptual salience, some speech sounds are *a priori* better candidates than others. Some, such as strident fricatives, trills, or clicks, may be intrinsically more conspicuous, perceptually speaking, than (say) back rounded vowels or nasals, such that irrespective of any social information these more prominent sounds might convey about the talker they simply stand out from the acoustic background more than other sounds do (‘bottom–up’ salience); in the parsing of the speech stream, more salient sounds, according to Goldstein (1977, p. vi), ‘constrain higher-level decision processes more than others,’ affording them special value as conduits for linguistically relevant information (see also, e.g., Narayan, 2008). The notions of markedness and frequency are implicated too, as Bardovi-Harlig (1987), Podesva (2006), Honeybone and Watson (2013), and Watson and Clark (2013) demonstrate. It seems prudent, in any event, to allow for a certain level of unpredictability, even arbitrariness, when it comes to the identity of speech features destined to become sociolinguistically salient, in view of the evidence showing that features apparently lacking much acoustic or auditory prominence can nonetheless acquire substantial sociolinguistic salience. An example might be ‘TH-fronting,’ whereby the English dental fricatives /𝜃/ and /ð/ are realized as [f] and [v] respectively. Miller and Nicely (1955, p. 347) find that under controlled experimental conditions the distinctions between the fricative pairs [𝜃]∼[f] and [ð]∼[v] seem especially difficult for listeners to hear reliably, and yet in contemporary UK English TH-fronting is a widely attested sociophonetic variable that has long attracted overt comment and at times considerable stigma from laypeople (e.g., Levon and Fox, 2014; Baranowski and Turton, 2015). Where there is little to distinguish speech sounds from one another acoustically, it becomes more challenging to identify reasons why listeners might treat the forms in question as more significant social information-bearing units than others that they hear.

As we have suggested above, the associations that listeners make between linguistic forms and speakers’ social characteris tics, and the extent to which listeners agree on those associations, vary from community to community. These sets of associations are therefore dynamic rather than static. We should also take account of how closely they are tied to production patterns in the communities to which the listeners belong, given that these patterns are similarly variable from place to place, and in view of the mutual dependence of production and perception. Our aim, then, is to assess salience in respect of the social category associations that linguistic forms embody for community members, as well as to examine correspondences between patterns in listeners’ perceptual responses to a form, and spoken productions of the same form within the listeners’ speech communities.

### The Operationalization of Salience in a Border Zone

Contexts in which markers of ingroup and outgroup status are known to be particularly prominent present ideal test sites for the investigation of salience. Such contexts can be found in border regions, where linguistic and non-linguistic markers of claimed and ascribed identities generally abound. These markers are described by Kiely et al. (2001, p. 33) as ‘[t]hose social characteristics presented to others to support a national identity claim and looked to in others, either to attribute national identity, or receive and assess any claims of attributions made.’ One of the behaviors that is most accessible to observers as a marker of this kind, according to Kiely et al. (2001), is accent. It follows that accent or dialect differences between localities in close geographical proximity to one another may be particularly sharply demarcated if the localities are separated by a political border.

The salience of linguistic features has an important function for the inhabitants of border regions, as it assists with the categorization of speakers as ingroup or outgroup members according to a superficially straightforward binary distinction: that of being from one side of the border versus the other. In certain cases, linguistic forms may mark speakers out as members of a transborder community in a zone which straddles the border and which is distinct in social and/or linguistic ways from regions further away from the border. But in either scenario, linguistic forms are key carriers of social meaning that pertains to national and regional identity groupings.

Even when movement across a border is not in any way impeded, a political border – by definition – marks a discontinuity of some kind. We can in many cases point to linguistic artifacts of the divide: there are numerous dialect isoglosses which coincide closely with political boundaries, for instance. When isoglosses bundle together like this, we can say we have evidence for a dialect boundary. Regions where marked accent or dialect differences exist, such as areas divided by political borders, have great potential in terms of their capacity to show us how those differences are exploited by members of the communities as a means of claiming or ascribing different national identities in casual spoken interactions. Clearly, there will be many features which contribute to the listener’s classification of an interlocutor as a member of a group from one side of the border or the other, but some features are likely to weigh more heavily in this evaluation than others. As border zones lend themselves very naturally to this kind of dichotomous grouping of speakers in terms of one nationality versus another, it is justifiable to treat linguistic variables which elsewhere may have complex and multiple indexicalities as forms which embody a binary opposition association (*nationality X* versus *not-nationality X*). We do so under the assumption that, in a border zone, this opposition is one that is both highly relevant and frequently encountered by local inhabitants. We are then in a position to put to the test our hypotheses concerning the extent to which people living close to the border share the perception that a form reliably marks one national identity but not the other, as well as to measure the strength – that is, the relative salience – of that perception within their communities.

#### The Scottish/English Border Region

We chose as the context of the present study four communities lying close to the political border separating Scotland from England. Inhabitants of localities in Scotland and England, two of the constituent nations of a single state (UK), have the possibility of claiming identities (*Scottish* versus *English*) which serve to distinguish them from people from the other side of the border, as well as an identity which unites them as a single category (i.e., *British*). This particular border therefore offers a productive testing ground for theoretical models of the convergent and divergent linguistic processes that take place along and across national and regional borders, and how these processes are manifested in the domains of speech production, speech perception and the claiming and ascribing of identity groupings.

Stretching for approximately 100 miles (160 km), the border separating Scotland and England is short compared to many other political frontiers. Nonetheless, its importance in linguistic terms is considerable. It has, indeed, been claimed to coincide with one of the most significant dialect boundaries in the Anglophone world. So numerous are the discontinuities in the distributions of phonological features in the area that the border has been dubbed a ‘strong linguistic barrier’ (Ihalainen, 1994, p. 248), while Aitken (1992, p. 895) asserts that the political border aligns with the ‘most numerous bundle of dialect isoglosses in the English-speaking world.’ This isomorphy, according to Aitken, effectively turns Scotland into a ‘dialect island.’ Among the phonological features that Aitken lists as contributors to the distinctiveness of Scottish varieties are the realization of the STRUT^1^ vowel as [Ʌ], the distribution and pronunciation of /r/, and the presence of the velar fricative [x] in words such as *night*. The Scottish Vowel Length Rule (SVLR; see Materials and Methods), a coherent set of alternations affecting multiple vowels in the system, is also seen as a key diagnostic of Scots and Scottish English (Aitken, 1984).

Glauser’s (1974) traditional dialectological survey of the region revealed that the political border also coincided with a substantial bundle of lexical isoglosses. Glauser surveyed 106 locations around the border by collecting data from one informant per locality. The most common type of isogloss Glauser recorded was one separating a dialect form on the Scottish side of the border from a non-localized or standard form used on the English side Glauser (1974, p. 278). When analyzed together, the isoglosses in Glauser’s survey clustered particularly densely in the central, upland stretch of the border, which then (as now) was much more sparsely populated than the areas at the border’s eastern and western ends. Transition zones were found at either end of the border, and while at the western end the transition zone straddled the border, in the east it occupied only the English side.

**FIGURE 1:**
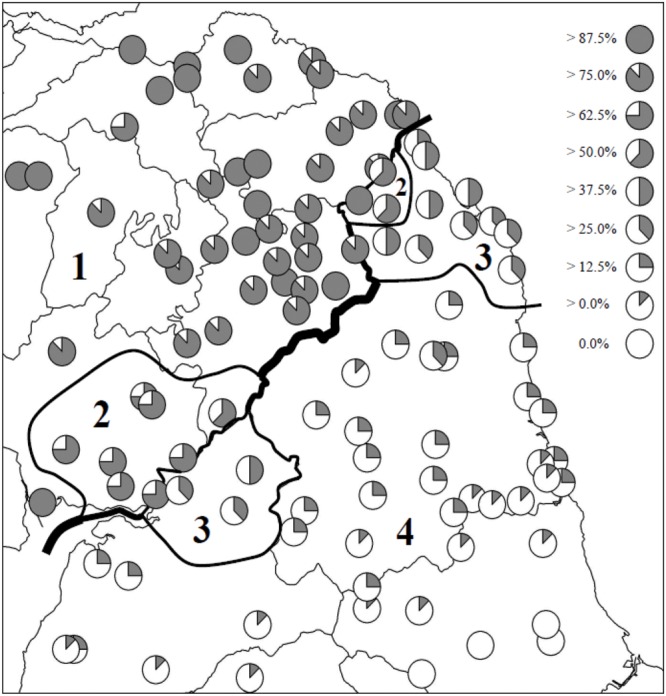
**Map showing percentage of ‘Scottish’ variants in traditional Scots and Northern English dialects (after Maguire, 2015, p. 447)**.

Of more relevance to the present study is Maguire’s (2015) examination of phonological differences in the traditional dialects spoken on either side of the border. By plotting 22 of the dialects’ phonological features, Maguire (2015) set out to investigate whether the same distributional patterns mapped by Glauser were also in evidence where phonological variation was examined. Of the phonological variables investigated, onset and coda /r/ were included, as was a vowel (PRICE) conditioned by the SVLR. For each locality, a ‘Scottishness’ index expressed as a percentage was calculated by pooling data collected from fieldwork sites sampled for volume 3 of *The Linguistic Atlas of Scotland* (LAS3; Mather and Speitel, 1986), the *Survey of English Dialects* (Orton and Dieth, 1962–1971), the *Orton Corpus* (Rydland, 1998), and unpublished data gathered for the *Linguistic Survey of Scotland*. **Figure 1** presents Maguire’s mapping of the degree of ‘Scottishness’ of the 22 phonological variables examined.

Maguire’s findings reveal a pattern very similar to that which emerged from Glauser’s lexical survey. A robust linguistic divide, more sharply delineated in its upland middle section than at its lowland endpoints, is resolved. Furthermore, the same transition zones – the western one spanning the border, the eastern confined to just the English side of the border – are visible in the phonological distributions. Evidence of the dividing effect of the border on the traditional dialects is clear: as Maguire puts it (Maguire, 2015, p. 448), ‘we have two independent studies which confirm that the Scottish–English Border is the locus of a significant dialect discontinuity.’

Studies documenting phonological variation in the border zone since the traditional dialectological work was carried out have observed the erosion of traditional dialect forms in favor of patterning of a less localized nature under the influence of the standard Englishes of both England and Scotland (see further Johnston, 1980). Even so, linguistic distinctions between the border localities persist, as research by Maguire et al. (2010), and McMahon and Maguire (2011, 2013) demonstrates. Using an algorithm that generates a cross-dialectal distance metric, six varieties spoken in the border zone were compared. In line with the results described above, the analysis yielded evidence of a sharp distinction between the dialects from Scotland and those from England. Rhoticity is found to be a major contributor to the similarity measure, such that varieties cluster more tightly according to whether they are rhotic or non-rhotic than they do in respect of other similarities. In spite of the attrition of many of the features of traditional dialect which served to differentiate varieties from either side of the border, Maguire (2015, p. 452) concludes that ‘modern accents in the Border area are as complex as was the relationship between traditional dialects of the early 20th century.’

In addition to the border’s continuing status as a major spatial discontinuity in the distributions of traditional lexical and phonological features in the region, a perception among non-linguists that the border represents a deep and entrenched linguistic faultline is also readily apparent. Perceptual dialectological research by Montgomery (2014) reveals that the border has a psychological effect on the perception of dialect areas, as evidenced through a map drawing task. Montgomery’s data, gathered from informants living in towns on either side of the border, demonstrated a unidirectional proximity effect, with his English participants showing relatively little knowledge of variation in dialects of Scotland by comparison with their Scottish counterparts. Among the latter group, knowledge of variation in dialects of English spoken in England was similar to that possessed by respondents from the English side of the border.

The discontinuities in pronunciation features that align with the border are evidence of the halting or slowing of the progression of various sound changes that have spread toward the border, principally from the south. Patterns of phonological variation in the region imply that local people have formed strong associations between these features and relevant social groupings based on prominent in-/outgroups. As the forms in question index particular social categories of relevance, their use persists for as long as it is in speakers’ interests to mark social category memberships using linguistic resources. The perceptual dialectological research undertaken in the area suggests that the border represents a psychological divide linked to the placement of accent groups. Although language in the area undeniably undergoes change, the border’s political and ideological implications are such that the view prevails that the border continues to represent a potent linguistic boundary. Indeed, Maguire (2015, p. 454) states that ‘[w]ith the transition from traditional dialects to modern accents, the Border is continuing to act as an important linguistic boundary, not watertight but certainly an impediment to change and indeed a focus of reinforcement of national identities.’

#### The AISEB Study

**FIGURE 2:**
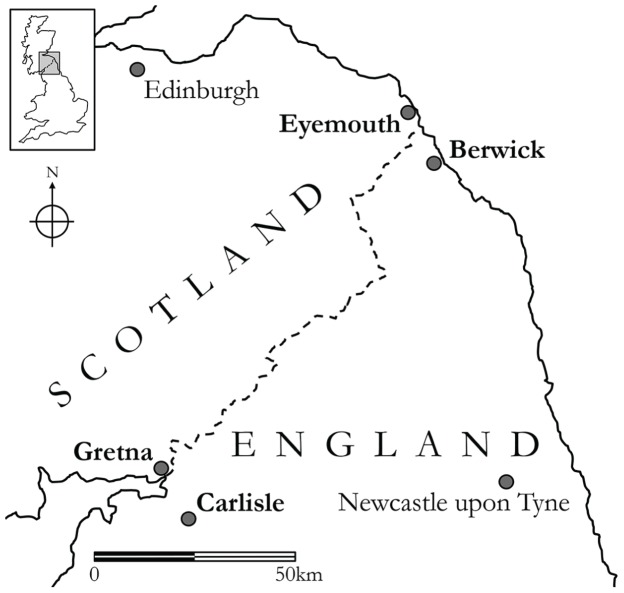
**Map of Scottish/English border region, showing the four fieldwork sites (labels in bold type)**.

The *Accent and Identity on the Scottish/English Border* (AISEB) project was an empirical investigation of phonological variation and change in four border localities and of the social-psychological effects of the border in terms of how ingroup and outgroup categorizations were constructed and enacted by people living in the area. Four fieldwork sites were chosen – Gretna and Eyemouth in Scotland, and Carlisle and Berwick-upon-Tweed in England (see **Figure 2**). We chose ‘paired’ communities lying very close to the border and to their partner locality: the distance between Gretna and Carlisle, and between Eyemouth and Berwick, is less than 10 miles (16 km). The two Scottish localities are considerably smaller than the English ones [Gretna (2,700); Eyemouth (3,400); Carlisle (107,500); Berwick (12,000)].^2^ The border does not inhibit movement – in physical terms it is invisible but for a few signs and flagpoles at the roadside – and in consequence there is plentiful contact between the paired cross-border localities. However, the population asymmetry in each pair of communities means that it is much more likely that residents of the Scottish towns will travel to the larger English localities than vice versa, a prediction which was confirmed very clearly in our informants’ interview responses. One might expect, then, that any linguistic changes taking place in the region would tend to go in the direction of the English model, with the Scottish speakers converging on the speech patterns of their English counterparts across the border.

The AISEB study took a tripartite approach to methodology, incorporating attitudinal and perceptual strands alongside the elicitation and analysis of production data. Our belief was that collecting data on the attitudinal positioning of the informants with respect to their identities and the socio-psychological effects of the border, and then combining these data with experimental evidence of the social meaning of key phonetic features, would yield a better understanding of the variation and change in the phonological patterns we uncovered. The current paper presents findings from just one of the tests used in AISEB’s perceptual strand, but relevant production and attitudinal data from the project are available in more detail elsewhere (see further Llamas, 2010; Llamas and Watt, 2014; Watt et al., 2014a,b).

As was found in previous surveys of the region (see The Scottish/English Border Region), production data collected for the AISEB project revealed marked differences between the Scottish and the English localities sampled. One of the main differences of particular relevance here was observed in rhoticity patterns. The speakers in the English localities were found to be effectively non-rhotic, while on the Scottish side of the border, rhotic forms were frequent. Eyemouth speakers, in particular, demonstrated near-categorical levels of r-fulness. In Gretna, at the border’s western end, rhoticity was much rarer and moreover was found to be decreasing considerably, with the younger speakers using markedly lower rates of rhoticity than the older speakers (around 15% versus approximately 45%; see Watt et al., 2014b for further detail). These findings are in line with those from other varieties of Scottish English, particularly in Edinburgh and Glasgow (e.g., Lawson et al., 2014), where the process of derhoticization (coda /r/ loss) appears to be well underway.

Contrary to Rácz’s assertion than coda /r/ is ‘entirely ignored by the speaker’ (Rácz, 2013, p. 147), we found that when our participants were asked to identify features associated with ‘Scottish’ as opposed to ‘English’ speech, they singled out /r/ more frequently than any other phonological feature in the border area, claiming it to be diagnostic of national and/or regional identity. Other pronunciation features were seldom mentioned, and were certainly not identified as consistently as /r/ is. Glauser’s (2000, p. 75) suggestion that variation in /r/ is of primary importance among the set of features that inhabitants of the border area use to categorize speakers as Scottish or English appears to us a very reasonable stance. On balance then, and in light of the divergent production patterns mentioned above, we hypothesized that /r/ – particularly where it occurs in coda position – is the phonological form with the highest degree of salience in the border zone. We turn next to the methods we used to test this prediction.

## Materials and Methods

The new and innovative SCAT formed part of a battery of tests designed to examine speakers’ perceptions of social category associations and of the geographical and social distributions of key phonetic forms in the border region. The tests were run on a subset of 40 of the original 160 speakers who had previously participated in the production and attitudinal strand of the project. For practical reasons, only a quarter of the full sample was invited to participate in the perception study, the time demands on individual participants having already been fairly heavy. The 40 subjects (10 in each of Eyemouth, Gretna, Berwick, and Carlisle) were split evenly by gender (male versus female) and into younger and older age groups (ages 16–24 and 57–82, respectively). The sample, therefore, can be divided into 20 older and 20 younger participants, as well as 20 Scottish and 20 English subjects. The fieldworker administered the perceptual tests in participants’ homes. The study was carried out with approval from the Ethics Committee, University of York, UK. All subjects gave written informed consent in accordance with the Declaration of Helsinki.

The SCAT ran as an adapted version of the IAT commonly used in psychological research (Greenwald et al., 1998). The IAT is typically used to access implicitly-held attitudes or associations by measuring the subject’s automatic associations between different target categories (e.g., Black people versus White people) and positive or negative attributes, represented by adjectives with positive or negative meanings (e.g., beautiful, annoying, etc.). A series of sorting tasks is used to assess the automaticity of association between the target categories and positive or negative attributes. The difference in response times when the target category is sorted with positive as opposed to negative attributes is taken as a measure of the difficulty of the task for the subject, and is argued to reveal differences in the subject’s implicit attitudes between the target categories.

For the SCAT, the framework of the IAT was implemented in *PsyScope* (Cohen et al., 1993). Audio samples taken from word list readings from the larger production study sample were played to subjects through headphones, and their task was to indicate, as quickly as possible, whether they associated the sample with either *England* or *Not England* in one of the test blocks or *Scotland* or *Not Scotland* in the other. Unlike the IAT, there were no right or wrong answers in terms of the sorting task; any response was considered valid. The speed of the subject’s decision and the degree of consensus across the group(s) to which the subject belonged were the metrics used to quantify salience in this experiment.

Single-word audio files containing each of the target forms were extracted from recordings of authentic Scottish and English individuals who had participated in the production strand of AISEB. The phonetic forms chosen for the audio samples were:

- /r/ in coda position – *car* [kɑɹ] (rhotic) versus [kɑ:] (nonrhotic)
- tapped or approximant realizations of /r/ in onset position – *red* [ɾɛd] versus [ɹɛd]
- short or long realizations of FLEECE – *need* [nid] versus [ni:d]
- front or back realizations of GOOSE – *spook* [sp
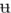
k] versus [spuk].

Although increasing derhoticization (also described as ‘/r/-vocalization’) has been reported in Scottish varieties since the 1970s (see Reid, 1978; Romaine, 1978; Macafee, 1983; Johnston, 1997; Stuart-Smith, 2003, 2008; Stuart-Smith et al., 2014, among others), rhoticity is still considered one of the critical defining features of Scottish varieties of English (Wells, 1982). Northern England is effectively non-rhotic (Beal et al., 2012), and it was clear by the 1970s that derhoticization in Northumbria, England’s northernmost county, was already practically complete (see Påhlsson, 1972). As noted in The AISEB Study, findings from the production strand of AISEB confirm this discontinuity, in that the speakers we recorded in the English localities were almost uniformly non-rhotic, while those on the Scottish side of the border continued to exhibit high degrees of rhoticity (see further Llamas, 2010; Watt et al., 2014b). As is also noted in The AISEB Study, important east/west differences were revealed in the amount of rhoticity found among Scottish speakers, a factor we consider further in East/West Cross-Border Community Pairings.

In onset position, the alveolar tap [ɾ] occurs in varieties of English spoken in both Scotland and the far north of England (Llamas, 2001; Johnston, 2007; Stuart-Smith, 2008), but among the varieties spoken around the border is very much more frequent in the Scottish ones (Watt et al., 2014b). The approximant realization of /r/ can likewise be heard on either side of the border, but is more frequently and consistently used by speakers in England by virtue of their near-categorical avoidance of the tap and other available variants, and indeed is associated with England to the extent that in his work on phonological variation in the border area Glauser (2000) refers to [ɹ] as the ‘English /r/’ in opposition to the ‘Scottish’ taps and trills.

The two vowel variables that we chose to include in the SCAT test, FLEECE and GOOSE, exemplify variation in quantity and quality respectively. The variants of FLEECE represent a difference of vowel length consistent with the durational conditioning that results from the operation of the SVLR (Agutter, 1988; Scobbie et al., 1999a,b; Pukli, 2004). The SVLR results in vowels that are phonetically long before voiced fricatives, before /r/ and before a boundary (including a morpheme boundary). Elsewhere, they are short. The SVLR operates alongside the ‘voicing effect’ (Chen, 1970; Lehiste, 1996) that is thought to condition vowel duration in all varieties of English, including Scottish ones. The voicing effect predicts that vowels preceding voiceless consonants will be phonetically shorter than vowels preceding voiced consonants. The SVLR, by contrast, takes account not just of the voicing of a following consonant, but also of its manner of articulation and the morphological structure of words. The vowel in the stimulus word *need* used in the SCAT is followed by a voiced stop consonant, predicting a phonetically short vowel in the SVLR-conditioned realization. Although evidence of complex context-conditioning of vowel length akin to the SVLR has been reported for locations south of the border (see, for example, Agutter, 1988; Glauser, 1988; Milroy, 1995; Krause, 1997; Watt and Ingham, 2000; Llamas et al., 2011), for our purposes we are testing perception of an association of the short FLEECE variant with Scotland rather than England. For clarity, we will refer to the variable henceforth as FLEECE, although it is in fact the sensitivity of listeners to SVLR-conditioned vowel duration alternations we are attempting to test here. It would have been possible, for instance, to have instead used GOOSE for these purposes, GOOSE being the other monophthong that exhibits SVLR conditioning the most markedly and consistently in Scottish English.

The variants of the GOOSE vowel in the present case were chosen to illustrate a difference of quality rather than of length, however. In Scottish varieties of English, GOOSE is realized as a close, central vowel transcribed as [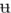] (Stuart-Smith, 2008), though it can also in fact be fully fronted. The North of England (particularly the North East), on the other hand, is one of an apparently dwindling number of places in the English-speaking world where close, back and fully rounded realizations of the vowel – i.e., [u] – can still be heard (Beal et al., 2012). The GOOSE item chosen for inclusion in the SCAT is *spook*, a word in which the vowel is predicted to be short in both English and Scottish varieties, as vowels preceding /k/ are exempt from SVLR-conditioned lengthening. Measurements of the vowel durations of the stimuli bear this prediction out, and the overall word durations also match closely.

We made every effort to ensure that the other characteristics of the stimulus words were as neutral and as closely comparable to one another as possible. That is, we checked carefully that there were no clear differences between the Voice Onset Time duration or degree of aspiration of /k/ in *car* (see e.g., Docherty et al., 2011), and that the vowel qualities in the two exemplars of this word matched closely. We detected no differences in the rhymes of the exemplars of *red* that might reinforce or confound listeners’ judgements of the stimuli based on the quality of the initial rhotic; neither did the consonants in our *need* and *spook* stimuli exhibit any dissimilarities that would concern us. The non-target parts of the test words are not absolutely identical, of course, but this is an unavoidable aspect of using natural stimuli rather than synthetic or spliced ones.

Based on previous literature, then, along with findings from the production strand of the research, the expected associations are those shown in **Table 1**.

**Table 1:** Expected associations based on previous literature and AISEB production data.

Scotland	England
Realized /r/ in coda	No realized /r/ in coda
Tapped /r/ in onset	Approximant /r/ in onset
Short FLEECE	Long FLEECE
Front GOOSE	Back GOOSE

Productions of forms predicted to be associated with Scotland were selected from recordings of informants from the Scottish localities Gretna and Eyemouth, while those predicted to be associated with England were taken from English informants from Carlisle and Berwick. All speakers were male, and were matched as closely as possible to one another for age and voice quality. Isolated tokens were drawn from word list readings, so as to ensure that all audio samples were clear and unambiguous. Listeners heard two forms of each target word (one rhotic and one non-rhotic token of *car*, one token of *red* beginning with the alveolar tap and a second beginning with an approximant, and so on), and were required to indicate using a computer keyboard the associations the forms evoked by pressing a key corresponding to the listener’s choice. The options the participants were presented with were the binary oppositions *Scotland*/*Not Scotland* or *England*/*Not England*.

As with the IAT design, the SCAT consisted of several blocks, and began with a practice block in which participants familiarized themselves with the layout of the computer screen and keyboard. The screen showed ‘PRESS ‘d’ FOR SCOTLAND’ in the top left-hand corner, and ‘PRESS ‘k’ FOR NOT SCOTLAND’ in the top right-hand corner. Either ‘SCOTLAND’ or ‘NOT SCOTLAND’ would then appear in the middle of the screen, and the participant had to press the relevant key as quickly as possible. The next block followed the same format, but this time the audio samples were introduced, and were accompanied by a visual representation of the stimulus word (a block of the color red and stylised pictures of a car, a ghost and a begging bowl for the words *red, car, spook* and *need* respectively). Participants, who listened to the samples through high-quality closed-cup headphones, were instructed to press either the key indicating ‘SCOTLAND’ or the one indicating ‘NOT SCOTLAND’ as quickly as possible after having heard a sample. This was also a practice block. The block that followed it was ostensibly the same as the practice block, but was the block from which the results were taken. For the fourth block the setup was again the same, but in this case ‘SCOTLAND’ and ‘NOT SCOTLAND’ were replaced by ‘ENGLAND’ and ‘NOT ENGLAND.’ As before, there was a practice block followed by the test block (block 6) from which results were taken. Half of the participants began with the *Scotland*/*Not Scotland* opposition and half began the SCAT with *England*/*Not England*, so as to compensate for any fatigue effects. Each sound file representing each variant was heard three times in random order in each block, making 24 stimuli in total per block (i.e., 3 repetitions × 4 words × 2 forms of each word). The keypress prompted the next screen and audio stimulus. In total, the six blocks of the test took between 5 and 10 mins for each participant to complete. As noted above, all participants had taken part in the earlier part of the study during which the production and attitudinal data were collected.

**FIGURE 3:**
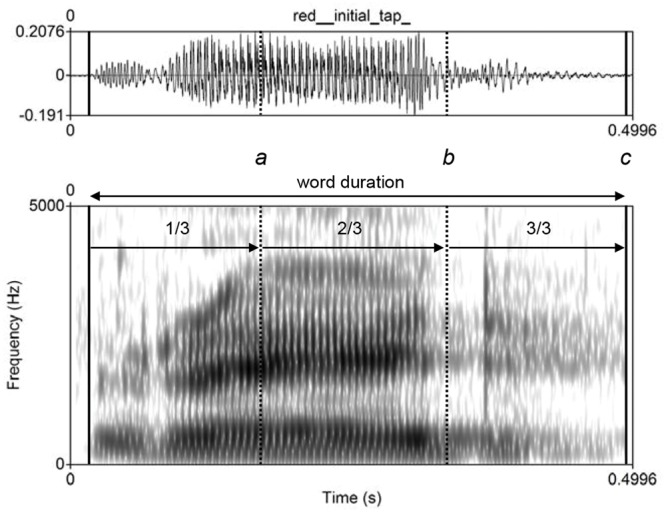
**Waveform and spectrogram of *red* produced with an initial alveolar tap [ɾ] by a speaker of Scottish English**. The superimposed solid vertical lines represent the start and end points of the word, while the dashed vertical lines dividing the word into three equal portions represent the alternative placements of the zero time point relative to which listeners’ response times were measured. Where the target form was word-initial (as in *red*), the response time was logged relative to point *a*, i.e., after one third of the word had elapsed; for a word-medial form (as in *need* and *spook*), the zero point was at *b*; and for word-final target sounds (as in *car*), the listener heard the entire word before he/she had the opportunity to respond (time point *c*).

Because the target forms appeared in different positions in the stimulus words, it was necessary to give listeners sufficient time to hear the form but also to respond to it as quickly as possible after exposure. We therefore adjusted the zero point from which the response time was measured depending on where in the stimulus word the target form appeared. Where the target form was word-initial, we allowed one third of the duration of the stimulus word to elapse before the zero point was reached. For word-medial forms, the zero point was placed two thirds of the way through the word, and for word-final forms, the zero point was placed at the end of the word (see **Figure 3**). In analyzing our results, as is common practice, we applied a lower cutoff at 200 ms to eliminate any values that were likely to be spurious. An upper cutoff at 3517 ms (=2.5 SD above the mean) was also identified. This resulted in a loss of only 2.6% of the data, a value falling well below the threshold recommended by Ratcliff (1993, p. 517), who advises that it is reasonable to apply a cutoff that eliminates not more than 15% of the total data.

The non-linguistic variables that were used to model the results were the listener’s age, gender, nationality, and the geographical location of his/her speech community of origin (East or West). The results were subjected to linear mixed effects modeling and logistic mixed effects regression in R, as appropriate.

## Results

The results of the SCAT are considered firstly in terms of the performance of each phonetic variant. We then turn to examine the influence of the listener’s social characteristics on how the social meaning carried by the form was perceived.

### Variation by Phonetic Form

#### Categorization

**FIGURE 4:**
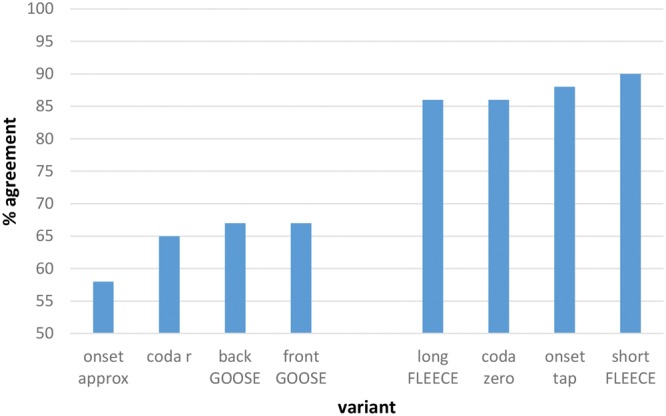
**Predicted levels of community agreement (%) on ‘correct’ association made between social category and phonetic form based on overall SCAT results.** Note that 50% represents chance level.

The degree to which the individual phonetic variants were more or less likely to be categorized according to the expected patterns was examined, and an overall model was initially run to test whether the phonetic variants had an effect on the predicted categorization. Using log likelihood comparisons, we compared a fully fit model with nationality, East/West, age, gender and phonetic variant as fixed effects to one without phonetic variant. The inclusion of individual phonetic variants significantly improves the power of the model (*p* < 0.001). A logistic mixed effects regression with phonetic variant as a fixed effect and individual participant and stimulus (word) as random effects was run. Onset approximant in the stimulus *red* (i.e., the pattern of responses for [ɹɛ]) was set as the baseline. **Figure 4** reveals the plot of the model’s predicted associations based on the raw data.

**Figure 4** reveals a cluster of forms that elicit high levels of community agreement with respect to associated social meaning, and a second group for which levels of agreement are substantially lower. These two clusters differ substantially for predicted levels of community agreement (*p* < 0.001). The onset approximant [ɹ] is the phonetic form with the lowest probability of being categorized according to the expected pattern (i.e., association with England). Indeed, categorization of [ɹ] is at around chance level, showing no association with one category more than the other. Describing the onset approximant as the ‘English /r/’, as per Glauser (2000), would therefore be misleading, and reflective of a view that is apparently no longer held by people from the border area, assuming that it ever was. Surprisingly, the r-ful coda realization (in [kɑɹ]) also falls within the low-agreement cluster. As noted in The AISEB Study, rhoticity was anticipated to be the feature with the highest degree of salience among the forms considered, yet it appears not to be marking an agreed social meaning of *Scotland* in the present case. Conversely, the non-rhotic form [kɑ:] falls within the high-agreement cluster in **Figure 4**, suggesting that this phonological environment is salient after all, even if the expected association of an approximant realization of /r/ in coda position and *Scotland* is not in fact agreed upon. It is possible that for these participants the use of the coda approximant [ɹ] is also associated with other varieties of English, such as American English, a variety to which participants are regularly exposed through the media. This lack of exclusivity might serve to dilute the association between coda [ɹ] and *Scotland*.

**Figure 4** also makes it clear that variants of the FLEECE vowel are highly salient, to judge from the level of community consensus about its social category associations. The phonetic variant that has the highest probability of being categorized according to the expected pattern is the short variant of the FLEECE vowel. The long variant is likewise agreed upon in the anticipated manner. The fourth form in the high agreement cluster is the onset tap (in [ɾɛd]). This appears to be highly salient in terms of agreement on its social meaning. Compared with the onset approximant, every other variant yielded statistically significant levels of community agreement on social category association according to the predicted pattern.

#### Response Time

The second measure used to estimate the salience of our target forms was response time. We expected that more salient forms would elicit faster responses than less salient ones. An overall linear mixed effects model was run with all phonetic variants included. As we did for testing categorization, log likelihood comparisons were run on a fully fit model with phonetic variant, age, gender, nationality, and East/West, and the same model without phonetic variant. The retention of phonetic variant significantly improves the power of the model (*p* < 0.001). Individual variant was entered as a fixed effect, and participant and individual stimulus (word) as random effects. Again, the onset approximant was set as a baseline.

**FIGURE 5:**
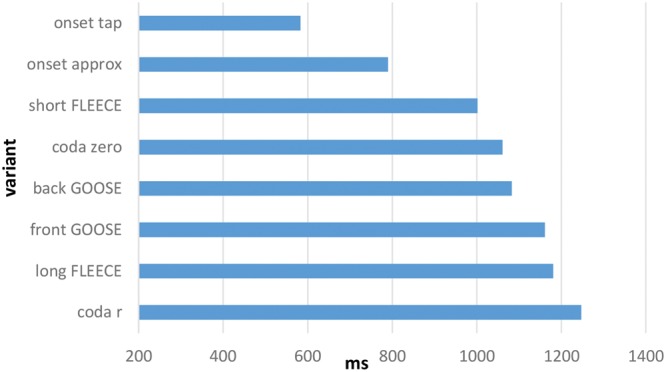
**Predicted response time (ms) to phonetic forms based on overall SCAT results**.

**Figure 5** is a plot of the predicted response times. It can be seen that the onset tap [ɾ] and onset approximant [ɹ] are reacted to faster than all other variants. The tapped form elicits an especially fast response time. **Figure 5** shows a marked difference between the response times for the variants of onset /r/ and the other variables, which cluster together in the ∼1000–1200 ms range (the difference in RTs between these clusters was substantial; *p* < 0.001). The difference between the results for onset /r/ and other variables suggests that participants possess a higher degree of certainty about the associations they make with onset /r/ than those they make with coda /r/ and the vowel variables. The slowest response time is found for coda /r/, suggesting a degree of hesitancy about the associations made with this form (cf. the discussion in Categorization, above).

#### Hierarchy of Salience

Ranking of each variant’s performance in the SCATs, as measured by community consensus and speed of association, reveals a hierarchy of salience. Taking both measures into account, we can say that the form with the highest level of salience among those examined is the tapped form of /r/ in onset position (as in *red* [ɾɛd]), given the very high level of community agreement on its association with *Scotland* and *Not England* combined with the speed with which the association was made. Variants of the FLEECE vowel are also imbued with a very high degree of salience as markers of Scottish versus English identities. The short variant of FLEECE and the *Scotland* association, contrary to our expectations, is the combination which predicts the highest levels of community agreement on social meaning. Non-rhoticity is also highly salient in terms of its agreed social meaning as a marker of *England* and/or *Not Scotland*.

Unexpectedly, the model does not predict high levels of agreement on the association of the r-ful realization of coda /r/ with any social category. Of the set of features examined, this is the feature that had been predicted to be the most salient. However, not only is there a lack of community agreement on its association, it also elicits the longest response time of all variants, suggesting even more strongly a degree of uncertainty around what social category it connotes. The other surprising result was the lack of association of the onset approximant with *England*, in spite of its treatment in the literature as the ‘English /r/’ (Glauser, 2000).

### Variation by Listener Characteristic

So far, we have considered the results of this experiment as though the participants were interchangeable members of a single monolithic community. We turn now to see whether age, gender, nationality (Scottish and English) or cross-border community pairing (East versus West) predict any differences in the reported degree of salience of the phonetic forms under investigation.

#### Nationality

In order to test overall rates of association according to the expected patterns, a logistic mixed effects model was run with nationality as a fixed effect and individual participant and stimulus (word) as random effects. Nationality was found not to be a significant predictor across all the variables when these were treated *en masse* (*p* = 0.107).

**FIGURE 6:**
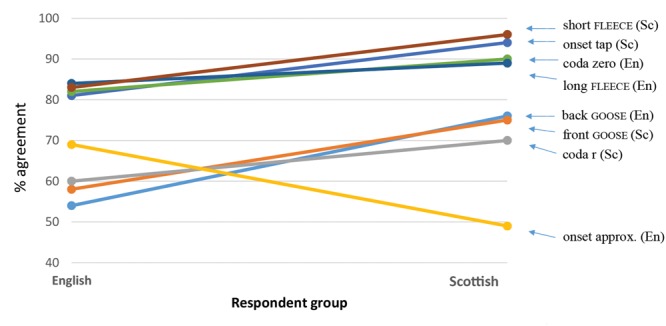
**Predicted levels of community agreement (%) on ‘correct’ association made between social category and phonetic form based on SCAT results from English respondents (left) and Scottish respondents (right)**. The abbreviations in brackets denote the ‘correct’ association. (Note that 50% represents chance level.)

Whether consensus of association across individual variants differed as a function of participant nationality was then tested by fitting a logistic mixed effects regression model with individual variant and nationality as fixed effects, and participant and stimulus as random effects. **Figure 6** is the plot of the model’s predictions.

**Figure 6** shows that for all but one of the variants under examination, the Scottish listeners are in closer agreement than are the English respondents about the ‘correct’ (expected) social category association made with the phonetic form. The only variant that fails to follow this pattern is the onset approximant (as in *red* [ɹɛd]), for which Scottish listeners are predicted to perform at around chance levels. English listeners are, by contrast, predicted to exhibit a moderate level of agreement where this variant is concerned.

As with the model based on the overall community results (see Variation by Phonetic Form), we see here a clustering of high-performing variants, and, although Scottish listeners perform more uniformly than English listeners in terms of community consensus, agreement about these forms (viz., variants of FLEECE, onset tap and r-less coda) is still very high, at over 80%, among English listeners.

#### East/West Cross-Border Community Pairings

**FIGURE 7:**
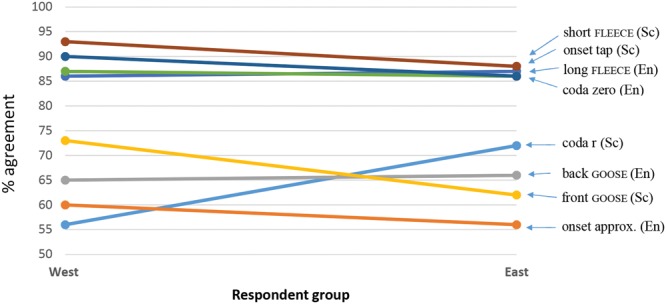
**Predicted levels of community agreement (%) on ‘correct’ association made between social category and phonetic form based on SCAT results from West respondents (left) and East respondents (right).** The abbreviations in brackets denote the ‘correct’ association. (Note that 50% represents chance level.)

Although the individual localities in the two pairs of communities (i.e., Gretna/Carlisle and Eyemouth/Berwick) are separated by the political border such that the nationality of participants from each of the four towns is a relevant factor, we can justifiably also view them as pairings which share the defining characteristic of being located at either the western end or the eastern end of the border. It seems natural to think that because they are both in Scotland the towns of Gretna and Eyemouth are somehow more similar to one another than they are to their respective nearby English partner communities on the other side of the border. But Gretna and Eyemouth, just like Carlisle and Berwick, are separated from one another by a relatively long distance, at least by British standards. Travel between the two same-nation localities along the length of the border is indirect and time-consuming even using private transport, so direct face-to-face contact between members of these communities is not likely to occur very often. By contrast, the conditions are very favorable for high levels of contact between inhabitants of the paired communities at either end of the border, in view of the fact that they live less than 10 miles (16 km) apart and experience no hindrances to their cross-border movement, as we noted in The AISEB Study. For these reasons, we turn now to a consideration of the two paired cross-border communities (Gretna/Carlisle and Eyemouth/Berwick) at each end of the border. **Figure 7** shows predicted differences between participants from the border’s eastern and western ends.

Despite the short distance between the two communities in each pair, and the separation of the same-nation localities lying at the border’s extreme ends, more difference is discernible between the respondents when they are classed by nationality than when they are grouped into cross-border communities. In terms of how they perform in the present experiment, the Gretna respondents have more in common with their fellow Scots in Eyemouth than they do with their English near-neighbors in Carlisle, for example. We do nevertheless see fairly close cross-border correspondences, particularly with respect to the high-performing cluster of phonetic variants. Where slight differences are in evidence, the tendency is for respondents from the western end of the border to categorize the target phonetic forms ‘correctly’ according to social meaning more often than is the case for those from the eastern end. There is one notable exception to this trend, however. Participants from the east are more likely to make the ‘correct’ association of overtly realized coda /r/ with *Scotland* than are their western counterparts. A logistic mixed effects regression model was fit with East/West and individual variant (fixed effect) tested as an interaction, and individual participant and stimulus as random effects. The presence of coda /r/ and a ‘correct’ association with *Scotland* was the only variant of the set to be affected by location (*p* = 0.034); there was no overall effect of East/West (*p* = 0.650).

This finding ties in closely with the production differences noted in The AISEB Study. With regard to the production of r-ful realizations, frequency of usage among Scottish speakers at the western end of the border (Gretna) is much lower than that recorded for the eastern Scottish (Eyemouth) speakers. In the AISEB sample, regular rhoticity is really only found in Eyemouth (see e.g., Watt et al., 2014b for further details of the production data).

#### Gender and Age

In order to test overall rates of association according to the expected patterns, a logistic mixed effects model was run with age as a fixed effect and individual participant and stimulus (word) as random effects. Age was found not to be a significant predictor across any of the variables (*p* = 0.857). Additionally, the effect of participant gender was tested for and was found not to be significant either as a main effect (*p* = 0.35) or as an interaction.

In order to test for whether participant age significantly affected response times, a linear mixed effects model with participant age as a fixed effect and individual participant and stimulus as random effects was run. The difference was not significant (*p* = 0.692). The predicted response time for younger participants was found to be 1003 ms, while for older participants it was 976 ms.

**FIGURE 8:**
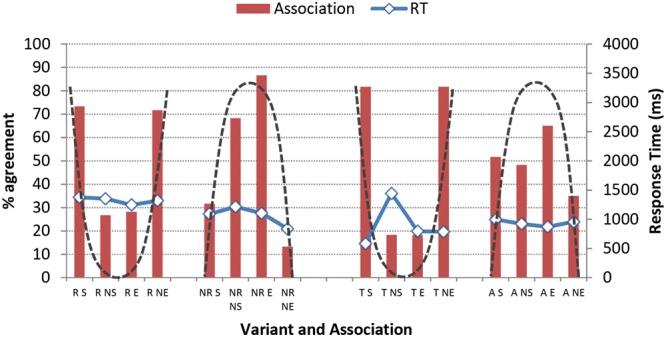
**Associations (bars) and Response Times (RT; solid lines) of older judges for variants of /r/.** (Variants are indicated as R = rhotic, NR = non-rhotic, T = tap, A = approximant; Social categories are indicated as S = Scotland, NS = Not Scotland, E = England, NE = Not England; Dashed line indicates shape of ‘correct’ pattern.) (Note that 50% represents chance level.)

**FIGURE 9:**
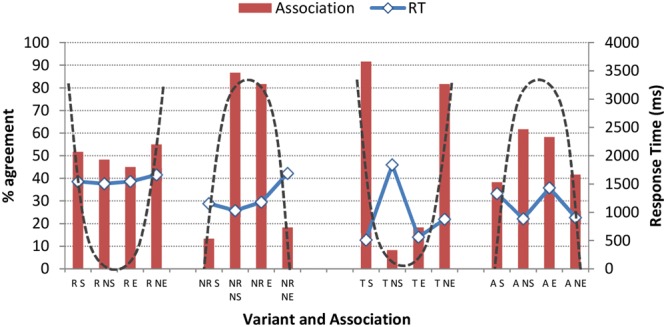
**Associations (bars) and Response Times (RT; solid lines) of younger judges for variants of /r/.** (Variants are indicated as R = rhotic, NR = non-rhotic, T = tap, A = approximant; Social categories are indicated as S = Scotland, NS = Not Scotland, E = England, NE = Not England; Dashed line indicates shape of ‘correct’ pattern.) (Note that 50% represents chance level.)

Although we found no significant effects for age overall, further inspection of the raw data revealed marked age differences in the degree of group consensus about association. **Figures 8** and **9** present the raw data for the associations listeners made with the two /r/ variables. The dashed line superimposed on each figure approximates the shape of the pattern predicted if a high proportion of ‘correct’ associations was made by listeners.

While the listeners’ agreement on the association made between tapped /r/ in onset position and *Scotland* remains stable across listener age, it is also apparent that the association with *Scotland* we expected to see when the listeners heard the rhotic form [kɑɹ] drops to chance levels, indicating that the association between r-ful realizations and the social category *Scotland* has become recessive. To test this hypothesis, a general linear model was run on the rhotic variant only, with participant age as a fixed effect. Younger participants were predicted to be significantly less likely to make the association between the rhotic form and *Scotland* (*p* = 0.001). They were, moreover, also predicted to be less likely to make the association between the non-rhotic form and *England* (*p* = 0.012). We know from the AISEB production data and the findings of other studies (see Materials and Methods) that derhoticization is underway in varieties of Scottish English, including the influential urban varieties of central Scotland, in that younger speakers produce fewer r-ful realizations than do their older counterparts. Here we see a loosening of the association of the r-ful pronunciation with Scotland, and a consequent diminution of the salience of the form.

Examination of the effects of listener characteristics has revealed that, in general, the patterns we observe hold across all listener groupings. However, the Scottish listeners in our sample are more likely to exhibit the anticipated associations between the high-consensus forms and the social categories *Scotland*/*not Scotland*/*England*/*not England* than are the English listeners. The other notable finding in the results broken down by listener characteristics is that perception mirrors production patterns, in the sense that the association between coda /r/ and *Scotland* – which we had hypothesized to be the strongest of any of the associations we set out to test – is weakening, just as overtly realized /r/ in syllable codas is becoming less frequent in Scottish English.

## Discussion

The approach taken in the present study rests, firstly, on the use of community consensus concerning the social categories that listeners associate with phonetic forms as a measure of the salience of those forms. Secondly, the speed with which subjects respond when making the association between a form and a social category is treated as an indicator of the association’s strength, and therefore of the degree of salience of the form in question. The results from the SCATs reveal that salience is a gradient property, such that salience-bearing forms can be ranked with respect to their relative salience. Certain forms, notably the short FLEECE variant and the realization of /r/ as the tap [ɾ] in onset position, are almost categorically associated with the social category *Scotland*. In the case of [ɾ], the association is made extremely quickly by listeners. Other forms, such as the onset approximant [ɹ], appear to possess negligible levels of salience.

The results presented above also demonstrate that salience is not a static property of phonetic forms or an inherent attribute of units of this kind. As we have seen, the degree of salience of a form, as estimated using measures of shared social meaning, can differ between speech communities separated by very small geographical distances, and also appears to change over time. Among other things, our findings strongly imply that a loosening of the association between r-ful pronunciations and the social category *Scotland* is underway in the region. Furthermore, the association of the r-less pronunciation with the category *England* robustly persists, demonstrating that the lack of a form can carry at least as much salience as its presence in equivalent contexts.

The SCAT results also demonstrate clear connections between linguistic production and perception. As noted above, our findings show a relaxation of the association between rhoticity and the social category *Scotland*, accompanied by a degree of hesitancy in making this association, as revealed through participants’ longer response times. These results coincide with changes in production patterns found in the larger AISEB study, whereby rhoticity appears to be decreasing rapidly in one of the Scottish localities (Gretna). In the broader context, we see that the process of derhoticization is well underway in the varieties spoken in Scotland’s dominant urban centers, Edinburgh and Glasgow (Stuart-Smith et al., 2014). This change appears to be most strongly linked to younger, working-class speakers. Mirroring these production patterns, we see in the results of the present experiment that, in terms of perception, the younger participants respond only at chance levels to the rhotic stimulus (*car* [kɑɹ]), demonstrating no agreement on its association with Scotland. We see further evidence of the interconnectedness of production and perception when we compare the results from the western end of the border to those from the eastern end. Western respondents are less inclined to make the ‘correct’ association than are their eastern counterparts. In the AISEB production data, levels of rhoticity are much lower for the western group than for the eastern group, and are decreasing over apparent time (see The AISEB Study), providing compelling evidence that the process of derhoticization is in progress in the west. Another example of the parallels between perceptual associations and production patterns is apparent in the lack of a strongly-held association between the approximant in onset position (in *red* [ɹɛd]) and the social category *England*. The high and increasing use of the approximant realization of /r/ in the Scottish localities is documented in the production strand of the study (e.g., Watt et al., 2014b). The reduction in the mutual distinctiveness of Scottish and English varieties brought about by this change in the distribution of [ɹ] is a probable contributor to the loss of the association of the approximant with England.

The findings of the present study, then, reveal a number of close links between production patterns and the perception of social meaning attached to a form. The salience of a phonetic form can increase or decrease depending on the usage patterns of the form. Thus, we would not argue that forms acquire salience, and remain salient thereafter, solely by virtue of their intrinsic phonetic properties. Rather, the strength of their socio-indexical value as seen through the lens of shared social meaning dictates how salient the forms will be. As production patterns change, so may the agreed social meaning of the form. Whether this is a direct causal relationship, or a bidirectional one whereby the one phenomenon acts as a trigger for the other, are matters for further investigation.

Finally, our findings lead us to sound a note of caution with regard to the prior assumptions that researchers bring to investigations of the sort represented in the current paper. Deciding in advance on which features are likely to have the strongest sociolinguistic salience in a given speech community may in general be inadvisable. Claims regarding the importance of /r/ to taxonomies of the subvarieties of English are abundant in the literature (e.g., Maguire et al., 2010, p. 97), and if we couple these claims with the frequency with which /r/ is mentioned as a stereotype of Scottish English by informants in the larger AISEB study, we could easily be led to form the expectation that the association between the r-ful pronunciation and the social category *Scotland* would be the most strongly-held association of those we tested. This prediction is, however, not borne out: in the statistical models, rhoticity was shown to yield *low* community agreement on its social meaning. As we noted earlier, the use of the approximant in onset position has been referred to as the ‘English /r/’ in this regional context (Glauser, 2000, p. 75), but a strong association of this nature is not observed in the results of the present study. We do, however, observe a robust connection between the non-rhotic form and *England*, so to this extent we do have evidence for the salience of (non-)rhoticity as a marker. Additionally, the use of tapped /r/ in onset position is extremely salient, according to the measures applied here. The phonetic feature which yielded the highest level of community consensus was, however, found to be the SVLR-conditioned alternation in the length of the FLEECE vowel, which is not a feature mentioned in any of the overt comments made by the AISEB informants.

The complex findings presented here clearly demonstrate the utility of the technique we used to collect them. The test we present here opens up new ways of investigating sociolinguistic salience. By using levels of community consensus about the association of phonetic forms and social categories as measures of the salience of the forms, we can posit a hierarchy of salience among key phonetic forms, and at the same time observe how features arrayed on this hierarchy may be re-ranked by members of the speech community in parallel with changes in production patterns.

## Conclusion

We have argued in this paper that, from a sociolinguistic perspective, the choice of features which become salient is in large part an arbitrary one. Salience depends on listeners initially noticing a feature and then collectively assigning social meaning to it. Under this definition, investigations of salience are examinations of perceptual aspects of the linguistic forms of which members of a given community or group have conscious or subconscious awareness (i.e., as stereotypes or markers, and indicators, respectively). Cognizance of a linguistic form may come about because the form is unusual in some way, and perhaps (but not necessarily) infrequent. It may also be occasioned because the form is an important marker of relevant ingroup or outgroup status within a speech community. It will only become an important marker of social category membership, however, if there is sufficient agreement among members of the speech community with respect to its function as a signal of group-membership meanings of this kind. Information about the association between phonetic forms and social categories among speech community members is usually not accessible via overt discussion. A way of operationalizing the salience of phonetic forms such that it can be empirically investigated, therefore, is by examining the extent to which the social meaning carried by the form, in terms of its social group associations, is shared by members of a speech community. This paper set out to test a method of estimating the relative salience of segmental variables, and has shown that not only is it possible to do so, it is also feasible using these techniques to examine the mutual dependencies between production, perception, and changes in salience over time.

Focusing on multiple localities in a border zone, a region in which social category divisions may be sharper and more prominent than in other places, enables us to see how phonetic forms are used to categorize speakers by social group, and permits us to identify those features which have sociolinguistic salience as group markers. Many linguistic forms are mobilized in the marking of social categories by speakers and listeners. Some forms, however, do more work in this regard than others. Comparison of levels of consensus about social category associations within and between communities, and of the speed with which these associations are made in the minds of listeners, gives us a means of estimating how salient a marker is relative to other markers. Estimating and tracking these changing levels of salience can then yield further insights into how and why language changes.

## Author Contributions

CL and DW designed the experiments, analyzed the data and wrote the manuscript. AM did the statistical modeling of the data.

## Conflict of Interest Statement

The authors declare that the research was conducted in the absence of any commercial or financial relationships that could be construed as a potential conflict of interest.

## Footnotes

1 Throughout this article we make use of Wells’ (1982) lexical set keywords, shown in small capitals (FLEECE, GOOSE, etc.), as a way of avoiding the ambiguity that often results from denoting vowel variables using symbols of the International Phonetic Alphabet.

2 Population figures are derived from Scotland’s Census Results OnLine (http://www.scrol.gov.uk/), Berwick-upon-Tweed Town Council (http://www.berwick-tc.gov.uk/town_council), and the UK Office for National Statistics (http://www.ons.gov.uk).
